# 1873. The role of electronic health record note templates to improve screening for tuberculosis risk factors in children

**DOI:** 10.1093/ofid/ofad500.1701

**Published:** 2023-11-27

**Authors:** Julia Fink, William Burrough, Charlotte Hsieh, Mariamawit Tamerat, Zarin Noor, Amit S Chitnis, Gena Lewis, Devan Jaganath

**Affiliations:** Lewis Katz School of Medicine, Temple University, Philadelphia, Pennsylvania; UCSF, San Francisco, California; UCSF, San Francisco, California; UCSF, San Francisco, California; UCSF, San Francisco, California; Alameda County Public Health Department, San Leandro, California; UCSF, San Francisco, California; University of California, San Francisco, San Francisco, California

## Abstract

**Background:**

Children should be evaluated annually for tuberculosis (TB) risk factors to detect and treat latent TB infection (LTBI), but there are gaps in screening and documentation. Embedding TB questions in an electronic health record (EHR) note template may increase screening.

**Methods:**

TB risk assessment questions were added to the well-child and -adolescent note templates at a Federally Qualified Health Center in Oakland, California in 2014. We extracted EHR data from all well patient visits from individuals 1-19 years old without a past medical history of latent or active TB over a 7-year period. We compared the proportion of visits that documented TB risk factor screening, before and after introduction of the note template and annually.

**Results:**

We examined 18,681 visits from 2014-2020, of which 7,071 (38%) were for children 1-4 years old, and 3,556 (19%) preferred a language other than English. One year after embedding TB risk factor questions in the note template, TB risk assessment increased from 76% to 92% (p = 0.02). In the subsequent six years, TB risk factor screening remained high ( >90%) and sustained (Figure 1). This trend was also seen among visits for those whose primary language spoken was not English. Screening for adolescents ≥12 years old improved over time (63% to 86%) but had lower overall screening than children 1-4 years old (≥90%) (Figure 1, p < 0.01).

**Figure:**
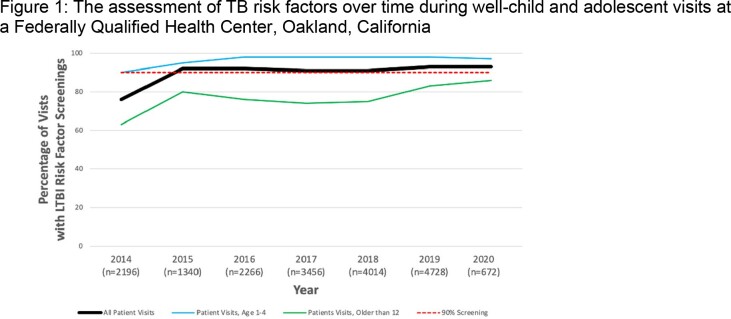

The lines represent the proportion of well-child or -adolescent visits that have documentation of a TB risk assessment, overall and for children who prefer a language other than English, as well as for children aged 1-4 years old and those older than 12. The red horizontal line indicates that assessments for TB risk factors were completed in 90% of visits during the given year.

**Conclusion:**

A simple EHR-based intervention can significantly improve screening and documentation for TB risk factors in the primary care setting and suggests that additional EHR solutions may address other gaps in the LTBI care cascade.

**Disclosures:**

**All Authors**: No reported disclosures

